# Applications of RNA Indexes for Precision Oncology in Breast Cancer

**DOI:** 10.1016/j.gpb.2018.03.002

**Published:** 2018-05-09

**Authors:** Liming Ma, Zirui Liang, Hui Zhou, Lianghu Qu

**Affiliations:** Key Laboratory of Gene Engineering of the Ministry of Education, State Key Laboratory of Biocontrol, School of Life Sciences, Sun Yat-sen University, Guangzhou 510275, China

**Keywords:** Precision oncology, Transcriptomics, RNA interference, microRNA, Breast cancer

## Abstract

**Precision oncology** aims to offer the most appropriate treatments to cancer patients mainly based on their individual genetic information. Genomics has provided numerous valuable data on driver mutations and risk loci; however, it remains a formidable challenge to transform these data into therapeutic agents. **Transcriptomics** describes the multifarious expression patterns of both mRNAs and non-coding RNAs (ncRNAs), which facilitates the deciphering of genomic codes. In this review, we take **breast cancer** as an example to demonstrate the applications of these rich RNA resources in precision medicine exploration. These include the use of mRNA profiles in triple-negative breast cancer (TNBC) subtyping to inform corresponding candidate targeted therapies; current advancements and achievements of high-throughput **RNA interference** (RNAi) screening technologies in breast cancer; and **microRNAs** as functional signatures for defining cell identities and regulating the biological activities of breast cancer cells. We summarize the benefits of transcriptomic analyses in breast cancer management and propose that unscrambling the core signaling networks of cancer may be an important task of multiple-omic data integration for precision oncology.

## Introduction

The fundamental mission of precision medicine is to confer the most appropriate management to patients within an appropriate time based on the clinical and molecular characteristics of their diseases [1], [2], [3]. The Precision Medicine Initiative was proposed in 2015, which consists of two main objectives, *i.e.*, a short-term goal aimed to improve cancer management, and a long-term vision promised to provide a better and healthier quality of life [4]. Oncology is considered to be “*the clear choice for enhancing the near-term impact of precision medicine*” [5]. Recent advancements in sequencing technologies and big data analytics have provided an unprecedented insight into the detailed molecular information of different tumor types, consequently promoting the development of potential targeted therapies and the innovation of clinical-trial strategies [6], [7], [8], [9], [10]. Comprehensive transcriptomic analyses present a global view of the RNA-based variants and contribute to the decoding of genomic data into actual gene expression patterns [11]. Transcriptomics is now regarded as an impactful approach to improving the application of genomic information to the identification, confirmation, evaluation and implementation in precision medicine exploration. In this review, we use breast cancer as a model to summarize the groundbreaking advancements and achievements of transcriptomics in cancer management in recent years.

**Figure 1:**
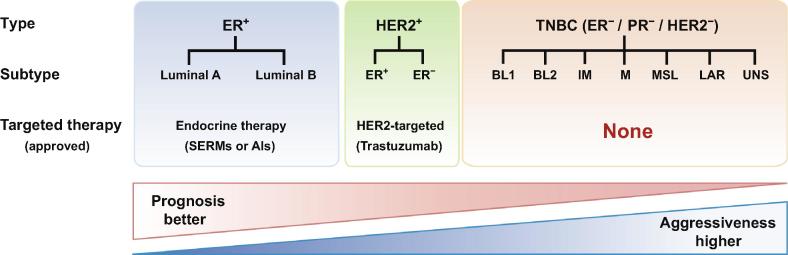
**Schema of the clinical classifications of breast cancers and the corresponding targeted therapies approved** Breast cancers are clinically classified into three types: ER^+^, HER2^+^, and TNBC, according to the expression status of ER, PR, and HER2, which can be further divided into several subtypes as illustrated. TNBC is associated with the worst prognosis and is more aggressive than the other two types, and currently there are no targeted agents approved for TNBC yet. ER, estrogen receptor; PR, progesterone receptor; HER2, human epidermal growth factor receptor 2; TNBC, triple-negative breast cancer; SERM, selective estrogen receptor modulator; AI, aromatase inhibitor; BL, basal-like; IM, immunomodulatory; M, mesenchymal; MSL, mesenchymal stem-like; LAR, luminal androgen receptor; UNS, unstable.

Breast cancer is the most common malignant tumor in women [12]. This extremely heterogeneous disease is clinically classified into three types (Figure 1), mainly depending on the expression status of estrogen receptor (ER), progesterone receptor (PR) and human epidermal growth factor receptor 2 (HER2). The ER^+^ group is the most common type of breast cancers (accounting for approximately 70% of all breast cancer cases), and endocrine therapies with selective ER modulators (SERMs) or aromatase inhibitors (AIs) have been adopted as the standard adjuvant treatments for ER^+^ tumors [13], [14]. The HER2-overexpressed group has achieved favorable therapeutic effects from the humanized monoclonal antibody trastuzumab that targets HER2 [15], [16]. Triple-negative breast cancer (TNBC) represents a distinctive subset of breast cancers with neither ER/PR expression nor HER2 amplification [17], [18]. TNBC accounts for approximately 15% of all types of breast cancers and is more malignant than the ER^+^ or HER2 highly-amplified breast cancers [18], [19], [20], [21], [22]. Current treatment of TNBC largely relies on chemotherapy and radiation therapy, with no targeted drugs approved for TNBC yet.

## Genomic architecture of breast cancer

The delightful advancements in whole-genome sequencing (WGS) technologies have provided an exhaustive description of the genomic landscape of breast cancer that includes rich information on DNA copy number aberrations (CNAs), driver mutations, and single nucleotide polymorphisms (SNPs) [23], [24], [25], [26], [27], [28], [29], [30], [31], [32], [33], [34], [35], [36], [37], [38], [39], [40], [41], [42], [43], [44]. A large number of CNAs, particularly deletions in *PPP2R2A*, *MTAP*, and *MAP2K4* genes, have been identified in primary breast tumors [28]. Using the highly multiplexed single-nucleus sequencing approach, a study involving 1000 single cells from 12 TNBC patients reveals that most CNAs are detected as early as the onset of breast cancer [35]. Inactivating mutations of *BRCA1* and *BRCA2* frequently occur in breast cancer as well [30], [36], [42], while unique mutations in *GATA3*, *PIK3CA*, and *MAP3K1* are enriched in the luminal A subtype of breast cancer [25], [45]. By analyzing the WGS data from 560 breast cancer samples, Nik-Zainal et al. further find numerous mutations in protein-coding genes [36]. TNBC exhibits a higher mutation rate than those observed in ER^+^ and HER2^+^ breast cancers, particularly in *TP53*, and an enrichment of the *MAGI3*–*AKT3* fusion is also detected in TNBC [24]. Notably, Ding et al. discover that the metastatic breast cancer shares 20 mutations with the primary tumor [23]. Yates et al. further confirm that the majority of mutations detected in the metastatic samples are similar to those present in the primary breast tumors, indicating that the metastatic clones probably arise from the primary tumors [38]. Additionally, two *ESR1* mutations (*ESR1^Y537C^* and *ESR1^Y537S^*) occur after the acquisition of endocrine resistance in response to long-term estrogen deprivation (LTED) [41]. In the last decade, genome-wide association studies (GWAS) have also discovered a series of novel breast cancer risk loci [29], [34], [39], [46], [47], [48], [49], [50]. These GWAS-identified loci contain abundant non-coding SNPs that could alter transcription factor (TF) binding sites and confer breast cancer-specific phenotypic variations [39], [51]. All of these studies have shed light on genetic susceptibilities to breast cancer and facilitated improvements in the prediction and assessment of breast cancer.

Genomic profiling has provided tremendously valuable information on genetic vulnerabilities in breast cancer; however, certain limitations remain. First, DNA variations may not reveal the actual activities of the corresponding biological pathways. In some cases, the master signaling pathways may be deregulated without any observed genomic alterations, and such cases are probably ignored in genomic analyses. Furthermore, even though the genetic variants have been discovered in genomics, it is often difficult to discriminate between “passenger” and “driver” mutations. Functional genomics needs to be replenished by large-scale data derived from other omic platforms. Transcriptomics is the most frequently used method for unscrambling genomic information [10], since it can comprehensively reflect the expression patterns of different kinds of RNAs [52], and is widely applied to investigate the genes that are differentially expressed under specific physiological or pathological conditions [53].

## Novel insights into breast cancer arising from transcriptomic analyses

### Technological advancements in transcriptome profiling

Transcriptomic analyses have been frequently utilized for exploring prospective biomarkers and potential therapeutic targets for human cancers [53]. Microarray analysis is useful in measuring the gene expression levels via complementary probe hybridization [54], [55], [56] and through which a large number of breast cancer-related genes have been discovered [57], [58], [59], [60], [61], [62], [63], [64], [65], [66], [67], [68], [69]. Furthermore, the wide application of RNA sequencing (RNA-seq) technologies has greatly expanded our knowledge of breast cancer [27]. Using RNA-seq, we can quantify genes that are expressed at extremely low levels and hence might be neglected when using microarrays [70], [71], [72], [73], [74]. RNA-seq also facilitates the analysis of specific fusion transcripts that are usually enriched in TNBC [24], [75], [76], [77], [78], [79]. More importantly, through manipulating RNA isolation prior to RNA-seq experiments, the transcriptome of specific ncRNA species, *e.g.*, microRNAs (miRNAs), can be enriched for better coverage [80], [81]. The applications of the transcriptomics to breast cancer studies are discussed below in more detail.

### Utility of mRNA expression patterns in TNBC subtyping

TNBC is exceedingly heterogeneous, poorly differentiated and highly metastatic [17]. It frequently afflicts young women and is associated with poor prognosis. Due to the lack of ER, PR, and HER2 expression, TNBC is insensitive to hormonal therapies. Therefore, conventional cytotoxic chemotherapy and radiation therapy remain the mainstay of treatment for TNBC, whereas the outcomes are far from satisfactory [12], [13], [14], [18], [19], [20], [22], [82], [83]. Efficacious targeted strategies are thus in urgent need for TNBC patients. Revising the sub-classification of TNBC into distinct molecular subtypes with unique transcriptional features is helpful for therapeutic decision-making and prognostic prediction [82], [83], [84], [33], [85], [86], [87], [88], [89].

Using massively parallel mRNA sequencing, numerous transcripts that are differentially expressed between TNBC and non-TNBC have been identified [83]. Based on the comprehensive transcriptomic analysis of 21 breast cancer datasets, Lehmann et al. classify TNBC into seven subtypes [82]. These include two basal-like subtypes (BL1 and BL2), an immunomodulatory subtype (IM), a mesenchymal subtype (M), a mesenchymal stem-like subtype (MSL), a luminal androgen receptor subtype (LAR), and an unclassified set that is regarded as unstable (UNS) (Figure 1 and Table 1). The BL1 subtype strongly expresses specific genes that are related to cell proliferation and DNA damage response. It preferentially responds to cisplatin and poly (ADP-ribose) polymerase (PARP) inhibitors. The BL2 subtype is enriched with genes that are associated with growth factor pathways, indicating that growth factor inhibitors may be efficacious for the BL2 subtype. The IM subtype possesses abundant genes that are involved in immune-mediated reactions, and programmed cell death 1/programmed death-ligand 1 (PD1/PDL1) inhibitors are anticipated to be a hopeful therapeutic option for this subtype. Both the M and MSL subtypes specifically express genes that are relevant to cell motility, cellular differentiation, and growth factor pathways, while the MSL subtype expresses lower levels of proliferation genes than those present in the M subtype. The mammalian target of rapamycin (mTOR) inhibitors and epithelial-to-mesenchymal transition (EMT)-targeted agents are candidate drugs for these two subtypes. The LAR subtype is named for the AR enrichment, and anti-androgen treatments (*e.g.*, bicalutamide, an AR antagonist) are undergoing clinical trials [82], [84], [89].

**Table 1:** **Transcriptomic subtypes of triple-negative breast cancer and potential therapeutic agents**

**Subtype**	**Percentage (%)**	**Cell line models**	**Unique pathways**	**Potential agents**
BL1	17	HCC2157, HCC1599, HCC1937, HCC1143, HCC3153, HCC38, MDA-MB-468	Cell cycle DNA damage response Proliferation genes	PARP inhibitors Cisplatin

BL2	7	SUM149PT, CAL-851, HCC70, HCC1806, HDQ-P1	Cell cycle DNA damage response Growth factor signaling	mTOR inhibitors Growth factor inhibitors

IM	18	HCC1187, DU4475	Immune signaling Cytokine signaling Antigen presentation	PD1/PDL1 inhibitors

M	24	BT-549, CAL-51, CAL-120	Cell motility Cell differentiation Growth factor signaling EMT	mTOR inhibitors EMT-targeted treatment

MSL	6	HS578T, MDA-MB-157, SUM159PT, MDA-MB-436, MDA-MB-231	Cell motility Cell differentiation Growth factor signaling Angiogenesis genes	PI3K inhibitors Antiangiogenic therapy Src antagonist

LAR	9	MDA-MB-453, SUM185PE, HCC2185, CAL-148, MFM-223	Androgen receptor Luminal gene expression	Antiandrogen therapy PI3K/mTOR inhibitors

UNS	19	HCC1395, BT20, SW527	Cell cycle DNA damage response Proliferation genes	PARP inhibitors Cisplatin

*Note*: Data are obtained from [82]. BL, basal-like; IM, immunomodulatory; M, mesenchymal; MSL, mesenchymal stem-like; LAR, luminal androgen receptor; UNS, unstable; PARP, poly(ADP-ribose)polymerase; EMT, epithelial-to-mesenchymal transition; mTOR, mammalian target of rapamycin; PD1, programmed cell death 1; PDL1, programmed death-ligand 1; PI3K, phosphatidylinositide 3-kinase.

### Functional characterization of breast cancer through RNAi screening

Genomic analyses have uncovered a rapidly growing number of genetic variants that may participate in cancer initiation and progression. However, two intractable challenges remain. On one hand, genomic analyses fail to distinguish between the “driver” mutations that are critical for pathogenesis and the “passenger” incidents that occur coincidentally. On the other hand, hundreds of unanticipated synthetic lethal (SL) interactions are hidden in cancerous abnormalities. SL interactions refer to gene pair relationships in which the separate inactivation of either gene does not affect the viability of cancer cells, but joint inactivation is lethal [10], [90], [91]. It is an intelligent approach to authenticating the inactivated genes first and then selectively inhibiting their SL partners to efficaciously kill the specific cancer cells. A well-known example is the use of PARP inhibitors in the management of *BRCA*-mutated breast cancer [92], [93], [94].

**Figure 2:**
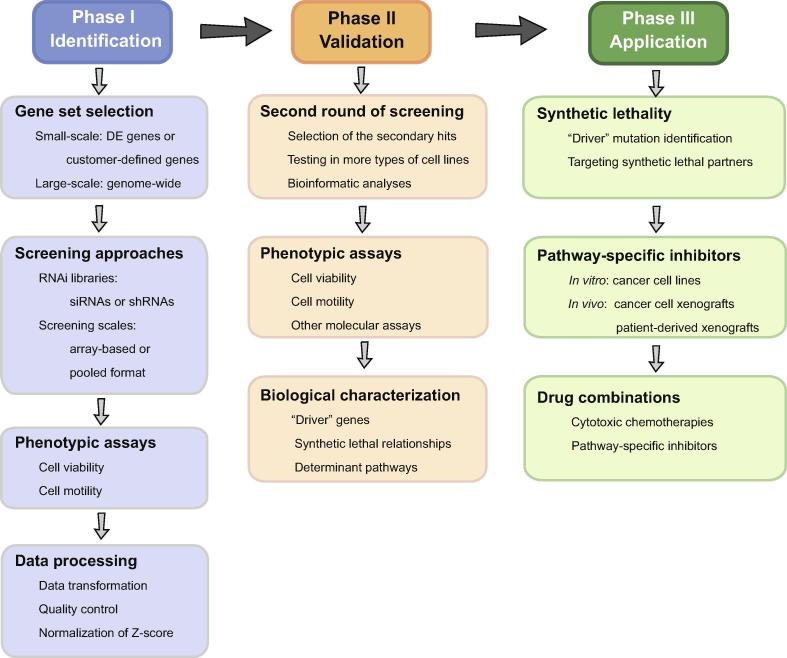
**A general flowchart for high-throughput RNAi screening** High-throughput RNAi screening usually comprises three phases. In phase I, the screening strategies, including gene sets, RNAi libraries, and screening scales, are determined mainly depending on the researchers’ purposes. The results of phenotypic assays are evaluated and normalized for the selection of effective hits. In phase II, the primary hits are validated by a second round of screening to confirm the “driver” genes, uncover the hidden synthetic lethal relationships, and disclose the critical signaling pathways. In phase III, targeted agents are tested both *in vitro* and *in vivo*, alone or in combination with other approved therapies. DE, differentially expressed; siRNA, small interfering RNA; shRNA, short hairpin RNA.

To circumvent the aforementioned limitations of genomic studies, loss-of-function RNAi screening technology has been widely adopted to define the functional genes that are necessary for cancer cells and to disclose SL relationships in exploring novel therapeutic options for cancer treatment [95]. Two types of RNA tools are used for RNAi, *i.e.*, small interfering RNAs (siRNAs) and short hairpin RNAs (shRNAs). siRNAs are applied to achieve transient and short-term gene silencing, whereas vector-based shRNAs enable stable and long-term gene silencing. Both siRNAs and shRNAs can be used in array-based screening or in pooled formats. Figure 2 presents a general flowchart for high-throughput RNAi screening.

In RNAi screening, a small-scale array can be used to selectively suppress the up-regulated genes that have been detected in previous transcriptomic analyses or the genes that are differentially expressed among different cancer subtypes [96], [97], [98], [99], [100], [101], [102]. Bauer et al. have made the first attempt to perform a vector-based shRNA screening targeting 428 genes that are derived from the overlay of a pool of abnormal transcripts in breast cancer and the druggable gene list. They find that inhibiting both *PPMID* and *SP1* significantly reduces the viability of two TNBC cell lines and increases their sensitivity to paclitaxel. When combined with paclitaxel, both CCT007093 and mithramycin, the respective chemical inhibitors of protein phosphatase Mg^2+^/Mn^2+^ dependent 1D (PPMID) and specificity protein 1 (SP1), suppress the growth of the paclitaxel-resistant TNBC cells [96]. In the same year, Kourtidis et al. have carried out a shRNA screen targeting 150 genes that are co-overexpressed with *HER2* based on previous meta-analyses and discovered that both *NR1D1* and *PBP* are novel survival factors essential for HER2^+^ breast cancer cells [97]. These two independent studies focus on two different types of breast cancers respectively, and uncover the distinct determinant genes between TNBC and HER2^+^ breast cancer. Subsequently, Marotta et al. further expand the number of breast cancer candidate genes and find that the IL-6/JAK2/Stat3 axis is significantly activated in CD44^+^CD24^−^ breast cancer cells [99]. In addition, two other groups perform siRNA screening by selectively focusing on the genes that are enriched in the aberrantly amplified regions in breast cancer, and identify several candidate oncogenic driver genes, such as *RAD21*, *EIF3H*, *CHRAC1*, *TANC2*, and *GNAS* [101], [102].

The development of large-scale RNAi libraries has enabled non-biased genome-wide loss-of-function screening [103], [104], [105], [106], [107], [108], [109], [110], [111], [112], [113], [114], [115]. Using high-throughput siRNA screening targeting the kinome, Brough et al. have defined a set of pharmacologically tractable genes in 34 breast cancer cell lines and uncovered the SL interactions between *PTEN* and *TTK* genes [103]. They further investigate the dependencies of kinase genes in ten cancers and utilize the resultant screening data to predict the drug sensitivity of the designated tumor cell lines by integrating with other molecular profiling datasets. They find that both *ERBB3* and *CCND1* are frequently amplified in breast cancer, whereas some skeletal system morphogenesis-related genes, such as *PDGFRA*, *ACVR2B*, *TGFBR2*, *DLG1*, *FGFR1*, and *FGFR2*, are highly-expressed in osteosarcoma [113]. In addition, using a pool of siRNAs targeting 17,378 genes, Petrocca et al. confirm that 154 genes are relevant to poor prognosis in breast cancer [107]. Marcotte et al. have conducted a genome-wide pooled screening containing 78,432 shRNAs of 16,056 unique genes in 72 cell lines for breast, pancreatic, and ovarian cancer. They discover that 297 genes are generally essential across all the cell lines examined [104]. Their further study on 77 breast cancer cell lines reveals that *BRD4* is a putative targeted option for luminal breast cancer and *PIK3CA* mutations probably determine the resistance to bromodomain and extra-terminal domain (BET)-inhibitors [112]. Moreover, by performing deep RNAi screening in 398 cancer cell lines, a recent study has identified a wide variety of cancer genes and constructed interaction networks among protein complexes and signaling pathways [95]. Taken together, these studies indicate that RNAi screening is a direct and impactful approach to identifying key determinants and informing novel therapeutic agents and drug combination strategies in breast cancer.

### miRNA signatures for TNBC

The majority of human genome, approximately 98%, is transcribed into ncRNAs [53], which consist of housekeeping ncRNAs and regulatory ncRNAs. The former includes rRNA, tRNA, small nuclear RNA (snRNA), small nucleolar RNA (snoRNA), and guide RNA (gRNA), whereas the latter includes miRNA, siRNA, piwi-interacting RNA (piRNA), and long ncRNA (lncRNA) [53], [71], [116]. miRNAs are well known for their involvement in various biological processes [117], [118], and a large number of miRNAs are deregulated in breast cancer [119], [120], [121], [122], [123], [124]. Using miRNA profiling in 31 primary TNBC cases and 13 lymph node metastatic samples in comparison with those from 23 matched normal counterparts, Avery-Kiejda et al. have identified 27 miRNAs related to the metastatic capabilities of TNBC cells [125]. Additionally, Koduru et al. have compared the publicly available small RNA sequencing data derived from 24 TNBC cases with those from 14 adjacent normal tissue samples and find that 55 aberrantly expressed miRNAs participate in the TGF-β signaling pathway [126].

**Figure 3:**
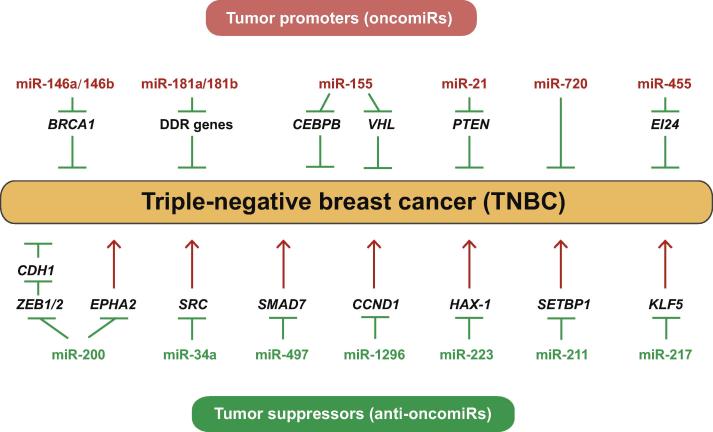
**Functional miRNAs involved in TNBC** Many miRNAs are differentially expressed in TNBC. They may act as tumor promoters (oncomiRs) or tumor suppressors (anti-oncomiRs) to regulate the capacities of proliferation and/or invasion of TNBC by suppressing their corresponding targets. DDR, DNA damage response.

The expression of some miRNAs is up-regulated in TNBC and these miRNAs may function as tumor promoters to increase the proliferation and/or invasion of TNBC cells. This type of miRNAs is thus termed as oncomiRs, which include miR-146a/146b [127], miR-181a/181b [128], [129], miR-155 [130], [131], miR-21 [132], [133], miR-720 [134], and miR-455 [135]. In contrast, the expression of some other miRNAs is decreased in TNBC and may function as tumor suppressors to inhibit cancer cell growth, induce apoptosis, and ameliorate metastasis. These miRNAs are termed as anti-oncomiRs, which include the miR-200 family [136], [137], [138], miR-34a [139], miR-497 [140], miR-1296 [141], miR-223 [142], miR-211 [143], and miR-217 [144]. These functional miRNAs and their corresponding targets are shown in Figure 3. Studies of the systemic delivery of miRNA mimics or inhibitors via nanotechnologies are ongoing and hold great promise for cancer management [145], [146], [147], [148], [149], [150], [151]. For example, RNA nanoparticles with an 8-nt sequence that is complementary to the seed region of miR-21 inhibit TNBC tumors in mouse models without affecting other healthy organs [149].

According to the critical roles of miRNAs in cell function and fate determination, a “Helm” model has been proposed to describe miRNAs as functional signatures for precisely characterizing cell identities in temporal-spatial specific status that primarily depends on the abundances of different miRNAs and the balance between these miRNAs and their corresponding targets [152]. In the case of breast cancer, comprehensive analyses of miRNA profiles combined with the mRNA expression patterns facilitate the illumination of the balance of miRNA-target pairs. We may be able to reclassify the breast cancer subtypes and clarify the unique biological capabilities of the selected cancer cells based on the dominant functional miRNAs.

## Conclusion and perspectives

**Figure 4:**
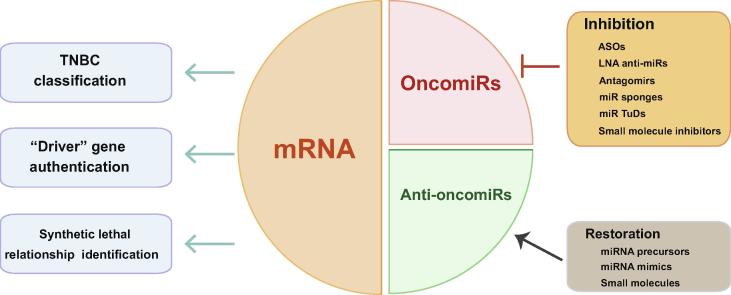
**Applications of mRNA and miRNA indexes in breast cancer** Transcriptomic analyses reveal the expression patterns of both mRNAs and miRNAs. TNBC has been classified into different subtypes according to the cluster analysis of the distinct mRNA expression profiles. Additionally, high-throughput RNAi screening is widely applied to authenticate the “driver” inactivated genes and identify the hidden synthetic lethal relationships. Transcriptomic analyses also facilitate the discovery and validation of breast cancer-associated miRNAs. Therapeutic strategies, based on the inhibition and restoration of deregulated miRNAs, are now undergoing trials and hold great promise for breast cancer treatment. ASO, antisense oligonucleotide; LNA, locked nucleic acid; TuD, tough decoy.

Transcriptomic analyses have provided massive amounts of information on the gene expression patterns in breast cancer. For clinical applications, the mRNA expression profiles can be employed to classify TNBC into unique molecular subtypes and to propose reliable therapeutic targets. Loss-of-function RNAi screening can be performed to discover the driver mutations and the SL partners of these inactivated genes for exploring novel targeted options. Moreover, an increasing number of miRNA are detected to be differentially expressed in breast cancer, many of which play critical roles as tumor promoters (oncomiRs) or tumor suppressors (anti-oncomiRs). The applications of transcriptomics in breast cancer are summarized in Figure 4.

**Figure 5:**
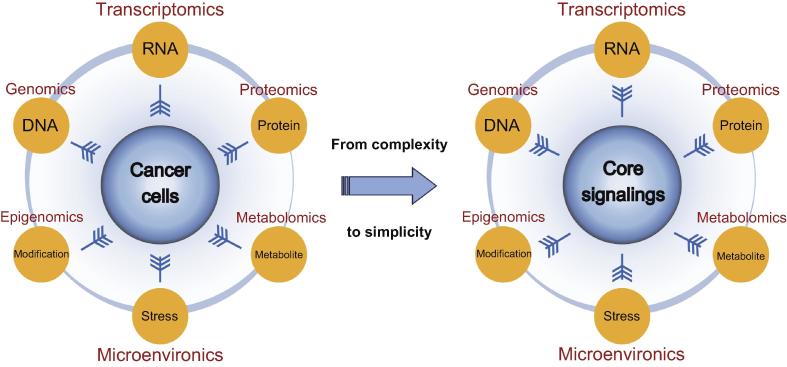
**Integration of multi-omic data to converge at the core signaling transduction pathways in cancer management** Comprehensive analyses of genome, transcriptome, proteome, epigenome, metabolome, and tumor microenvironment delineate the diverse aspects of cancer biology. Integration of these multi-omic data into the core intracellular signaling transduction pathways helps to provide accurate guidelines for cancer diagnosis and treatment.

With the development of high-throughput sequencing technologies and computational analysis tools, it has become much easier to obtain and decipher enormous datasets that are relevant to different biological layers of human cancers besides genomic and transcriptomic studies. Epigenomic studies reveal the architecture of epigenetic alterations in human genes, including DNA methylation and chromatin modifications [153], [154], [155], [156], [157]. Proteomic [158], [159], [160], [161], [162], [163] and metabolomic [164], [165], [166] studies are also useful for elucidating additional faces of cancer biology. Novel strategies for integrating the genomic, transcriptomic, epigenomic, proteomic, and metabolomic data are demanded for a holistic understanding of tumor evolution and development [167], [168], [169], [170], [171]. In addition, cancer cells are not isolated entities; rather, they communicate with other stromal cells and adjacent tumor sub-clones. The influence of the tumor microenvironment has come into public notice in recent years [172]. It is important and necessary to take the interplay between cancer cells and their microenvironment into account to understand the effects of external stresses on cancer initiation and progression, as illustrated in Figure 5.

The generation of multi-omic data has become an addictive routine for cancer studies. However, an intractable puzzle arises, that is, it is becoming increasingly intricate to assimilate the rapidly growing number of “big data”, as mentioned by Dr. Weinberg [173], [174], [175], [176]. Intelligent utilization and management of these data require massive computational resources and accurate statistical methodologies to unearth the hidden links among different subcomponents [174]. Although several data integration algorithms and a panel of software tools have been developed [177], there is still a lack of an impactful paradigm to solve Weinberg’s puzzle of how to effectively integrate multifarious information on cancer biology [173]. Multi-omic data display the myriad layers of cancer biology in detail, but the endless complexity seems to confuse our vision of the nature and the “Achilles’ heel” of cancers. Moreover, extreme inter- and intra-tumor heterogeneities originate from the rapid evolution of tumor cells. They may lead to a concept of personalized medicine for cancer therapy, which is based on every individual difference, rather than a concept that tumors are substantially a class of diseases that can be divided into several well-defined categories. We may return to simplicity and attempt to solve the complicated problems by identifying the common master regulators that correspond to the key hallmarks of cancers (*e.g.*, the capabilities of proliferation, metastasis, immune evasion, and energy metabolism) in different malignant cells.

We suppose that the core signaling networks that are derived from hundreds of unique cancer-related signaling transduction pathways may be the appropriate candidates to unravel Weinberg’s puzzle. These networks reflect the cell identities, including the varieties of biological capabilities and the temporal–spatial status in cell differentiation, and may be used as functional classification criteria for the characterization of different subtypes of human cancers. Accordingly, pathway-targeted drugs and therapeutic strategies may be precisely designed to redress the deregulated networks. The integration of multi-omic data into the core signaling transduction channels that determine the development of cancers may be a potential direction for us to go out from Weinberg’s puzzle of the biological “big data” and helps to provide accurate guidelines for diagnosis and management for cancer patients (Figure 5).

Breast cancer is well-classified using both cellular and molecular features. Titanic efforts have been made to acquire a great deal of multi-omic data that display diverse biological signatures of the development of breast cancer, particularly in TNBC, which has not been well-characterized and for which no targeted drugs are yet available. It is high time for us to take advantage of these rich resources to identify the core signaling networks in TNBC and we are filled with hope that this most difficult-to-treat cancer will be precisely targeted in the near future.

## Competing interests

The authors declare no competing interests.
